# Physiological Observations on the Transformations of the Organs of the Human Body

**Published:** 1806-12

**Authors:** C. L. Dumas

**Affiliations:** the Imperial Institute, Professor in the School of Medicine at Montpellier, &c. &c.


### *Physiological Observations on the Transformations of the Organs of the Human Body. By* C. L. DUMAS, *of the Imperial Institute, Professor in the School of Medicine at Montpellier, &c. &c.*

( Concluded from our last, pp. 451—455. )

An intelligent young physician has communicated to me a very interesting example of a transposition of the parts forming the articulation of the humerus and scapula. In this case, the glenoid cavity was wholly wanting, and

 from from the superior and anterior angle of the scapula proceeded a head similar to that of the humerus with its bicipital fossa and its cartilages. The humerus itself was enlarged towards the upper extremity, in which was a cavity like that natural to the scapula, notched towards its posterior edge, and which articulated with the head already mentioned. There was indeed a complete inversion of all the parts concerned in the formation of this articulation; on examining the opposite shoulder, the same organization was observable. The muscles, tendons, and ligaments exhibited nothing unusual in their appearance. I shall not here further insist on these singular aberrations of nature in the external and obvious structure of our organs, since they ought rather to be considered as a deviation from the usual law of organization, than as examples of degeneracy or degradation. The transformations of the organs relative to the internal disposition and arrangement of the parts, which enter into their more immediate structure, appear to be of much greater importance and utility to the physiologist and physician than the former. The accurate knowledge of these transformations leads us to suppose that the internal organization of the solid parts of the human body is neither so complicated, nor obscure, as is generally imagined. In this inquiry, our first object must be to determine, what are the principal classes of structure by the combination of which are produced all the different modifications of texture apparent on the dissection of dead bodies. On considering attentively the differences manifested in the arrangement and disposition of the substances composing the human body, they may I think be comprehended under six principal heads. The succession, or union of these dispositions in different organs determines the species of transformations of which these different classes of structure are susceptible. But before entering upon this explanation, it is necessary to form an accurate idea of the organized substances which are to be the object of it.

1st. The medullary or pulpy disposition constitutes the first class, and the most simple degree of structure. The medulla of the brain, the pulp of the nerves, the spinal marrow, the membranes, and the nervous papillæ furnish examples of it. This substance, though differing very little from the nature of fluids, yet possesses sufficient characteristic differences to entitle it to be ranked among the solid organs.

2d. The areolar or spongy disposition is comprehended under under the second class of structure; examples of which are furnished by the cellular and other membranes.

An idea may be formed of the areolar structure, by attending to the distribution of the cellular substance, in layers, between the fasciculi of the muscular fibres. In proportion as we separate these fibres from each other, it becomes elongated in the form of whitish laminæ, connected together by minute parallel filaments, intersecting each other, so as to leave more or less space between them.

It is truly surprizing to observe the tenuity of these laminæ and filaments, which by their irregular union form the cellules of this permeable tissue; of which the organization seems, as it were, only sketched. The approximation of these minute cells augments its density, in which respect alone, the structure of the serous membranes, or other similar parts, differs.

3d. The fibrous disposition comprehends the third class of structure; it is produced by the assemblage of fine, solid, and long filaments running in various directions, as may be observed in the structure of different muscles. These filaments, which are disposed in layers, separated from each other by means of the cellular tissue, and united into fasciculi, form fleshy masses, which are placed on the external surface of the body, as well as in the interior of the great cavities. These filaments adhere so closely to each other, as not to leave the smallest empty space between them. Each fibre detached from the general mass, may be subdivided into smaller filaments, until they become wholly imperceptible; and this division might be carried to a still greater length, did we possess instruments of sufficient delicacy for the purpose.

4th. The fibro-cellular disposition appears to constitute a fourth class of structure, under which may be comprehended the tendons, aponeuroses, periosteum, dura-mater, and all the membranes whose texture is wholly fibrous. A careful examination of their structure, evinces that they occupy a middle place, between the fibrous texture of the muscles, and the spongy tissue of the cellulur organs, and that they participate of the quality of this double structure, which is formed by a mixture of fibres and laminæ, combined together, and which only varies in the different organs, in proportion as one or other of these dispositions appears to predominate. In fact, the organization of the tendinous, aponeurotic membranous organs, is sufficiently different from that of the muscular organs, to prevent them being confounded with each other. There is nevertheless, a sufficient degree of similarity betwixt them, to warrant us to conclude, that they partake of the same nature. On the other hand, the relation which they bear to the cellular tissue, is not less obvious, since their fibres are capable of being converted into a similar texture, and they undergo the same change, when subjected to the process of maceration. Hence it is evident, that this is a mixed species of organization, depending on a particular mode of combination, in which the fibrous and cellular structures are so intimately combined, that we cannot perceive the distinguishing features of either. The membranes of the arteries and veins, as well as the external covering of the nerves, exhibit nearly a similar structure.

5th. The granular or parenchymatous disposition, constitutes a fifth class of structure. It is of a more intricate and complicated nature, than any of the former, and appears to include the three-fold organization of the pulpy, the cellular, and the fibrous substances. The different glands and viscera, such as the pancreas, the liver, spleen, &c. furnish examples of it. On a superficial view of the organs, partaking of this complicated structure, we merely observe an irregular, dense, shapeless, and fleshy mass of different colours; but on a closer inspection, we readily perceive that they are composed of a multitude of firm round granular follicles, connected together by loose cellular membrane, collected into distinct lobes, and inclosed in a common parenchymatous substance. Many anatomists believed that this structure was produced by the effusion of a nutritive coagulable fluid, while others compared it to a fine spongy cellular substance, the interstices of which had been filled up by concreted blood. The question was for a long time violently agitated; whether such organs were of a vascular or follicular structure; and it was only after much investigation, that the respective parties, who espoused these opinions, became convinced that though apparently opposite they might be reconciled with each other.

6th. The cellulo-calcareous, or lamellar disposition, constitutes the sixth class of structure, which is particularly observable in the cartilages and bones. Two very different substances concur in the organization of the bones: the one dense, hard, and solid, abounds chiefly in the middle portion of the long bones, and is termed the *compact substance*; the other spongy, open, and cellular, is chiefly found at the extremities of these bones, and is named the

*spongy*

*spongy substance.* A third, equally open as the second, surrounds the osseous cavities, and is known by the appellation of the *reticular substance.*

It is of importance to remark, that these three substances are originally formed of the same matter, and may all be separated into thin, soft, flexible, and cellular plates in infancy, when the whole of the bones constitutes only one mass, the structure of which appears in every respect similar to that observed at the extremities of the long bones in adults; whence I conclude, that bones are not composed of fibres or vessels, as is commonly taught, but of many laminæ and cellular fibres, more or less incrusted with a saline-calcareous matter, in proportion to their hardness and solidity.

The six classes of structure which I have enumerated, comprehend all the varieties of organization observable in the simple and regular substances of which the human body is composed. But there are others which cannot be reduced to any of these classes, because they are of a more intricate and complicated texture. Such, for example, is the reticulum mucosum, lying between the epidermis and the skin, which probably consists of a collection of capillary vessels, disposed in the manner of net-work; such also are the epidermis, the nails, the enamel, the hair, &c. which being feebly organized, enjoys but a small portion of vitality.

Though the structure of our organs be in general fixed and invariable, their organization and intimate texture may nevertheless be changed by disease, or other accidental circumstances. This change may occur between organs of the same texture; of which the difference is solely occasioned by the condensation or approximation of the parts which compose them. Thus the cellular tissue, when condensed, becomes a solid body, and analogous to the serous membranes; and these last, when dilated, assume the appearance of the areolar texture, common to the cellular organs. The spongy portion of the bones is frequently converted into a compact substance, while the more solid parts in their turn, assume the spongy texture. The tendons and aponeuroses, sometimes undergo a reciprocal conversion. The ligaments also become at times flattened, and acquire the appearance of fibrous membranes, while these membranes in their turn, assume the shape and form of ligaments. It is likewise not unusual to observe such a conformity between the parenchymatous substance of the liver and spleen, that they may readily be mistaken for each other.

The

The second class of transformations regards those organs, having an analogous conformation, but which differ by the addition, or substraction, of some part, essential to their structure. The analogy of simple substances, with those which are more complicated, renders such kind of transformations not unfrequent. They occur either when the texture of a part, as the cellular, for example, becomes more complicated, in consequence of being combined with the elements of another organ, or when the more complex textures, as the fibro-cellular and parenchymatous, become simplified and resolved into their elementary parts. We might here mention the conversion of the cellular organs into a parenchymatous substance, similar to that of the viscera. It has been already observed, that the substance of the lungs sometimes acquires the organization of the liver. I have myself observed in the cellular portion of the peritoneum, a species of irregular bodies divided into little lobes, having the structure and form of glands. The medulla of the brain is subject to two different kinds of transformations; in the first, it loses the small degree of solidity, peculiar to it in a healthy state, and is reduced to the consistence of an albuminous fluid; in the second, it gradually becomes condensed, and augments in hardness. The combination of the cellular and fibrous texture, andthe union of phosphate and osseous matter, constitute an organ equally firm and hard as flesh and bones.

The contrary action of the same causes, may decompose the texture of certain organs, and reduce them to the elements of which they are originally composed. The fibro-cellular membrane of the pericranium, sometimes exhibits the appearance of a simple cellular web, and at others of a large band of fleshy fibres, which may be detached from the cranium with the skin. The dura-mater inflates, thickens, dilates, and sometimes becomes wrinkled like the mucous membranes. The viscera and the glands, when deprived of a considerable portion of their parenchymatous substance, exhibit, instead of their natural texture, in one place, the appearance of a soft pulpy body; in another, a mass of spongy substance; and in a third, an assemblage of vessels and fibres. The bones, when deprived of their calcareous phosphate, return to their original cellular and cartilaginous texture. The original substances of the organs, such as the cellular and fibrous, may either enter into a perfect combination, which assimilates them together, or into a simple union, by which they aproximate and become connected with each other. On the combination of of two or more substances is established the compound structure of certain organs, which, like the viscera, the glands, the fibrous membranes, and the bones, retain no analogy or resemblance to the elementary parts from which they are derived. The union of these different classes of structure belongs to the organization of the muscles, in which the cellular membrane and fleshy fibre afford an example of two distinct substances, existing separately in the same organ. The transformation of the organs, which are produced by the mixture and juxta-position of their substances, is no where better displayed than in the singular transposition of the cellular and serous membranes into the state of muscular and fleshy bodies. We might here adduce the testimony of almost every anatomist, respecting this species of conversion, but it will be only necessary to cite the authorities of Reisselius, Lower, Bonnet, Vicq D'Azir, who witnessed in several dead bodies the pericardium, the peritoneum, the external membrane of the intestines or of the bladder, furnished with dense red fibres, so exactly similar to those of fleshy parts, that they appeared to be converted into muscles. But if nature, by adding the fibrous matter to cellular parts, can convert them into muscular organs, it seems probable, that by pursuing a contrary procedure, and suppressing the fibrous matter of muscles, she may in like manner, transform them into cellular substances. I perfectly recollect, that once, when dissecting a dropsical subject, I discovered several muscles, whose fleshy fibres had been destroyed, leaving only a mass, full of whitish cells, stamped with their impression.

The transformations of parts, which, though different, are yet similar in their chemical composition, might be considered as constituting a third class, if they were not more naturally included among the changes, relative to the matter and constituent principles of organs, which form the object of the second class. As the order, the developement, and proportion of these principles vary, they may give rise to numerous changes, so as to transform the very texture of the parts of which they supply the elements. In this way, cartilages assume the osseous character, while bones approach to the nature of cartilages. To produce this effect, it is only necessary that the proportion of the calcareous phosphate or gelatine be augmented or diminished, as has been shewn in the first part of the present essay.

Lastly, there are parts, which differ so with respect to structure structure and composition, that the transition from one to the other, appears to be accomplished, not by a real transformation, but by the successive developement of a texture, foreign to the natural state of the organ, which thenceforth assumes a two-fold structure. The dura-mater sometimes exhibits, from the numerous vessels scattered through its substance, all the characters of a vascular organization. This example, among several others, tends to determine, what constitutes the last genus of transformations, which depends on the structure of the organs.

The fourth and last class of transformations comprehends all those singular changes in which one organ supplies the place of another in the exercise of its vital properties and functions. This subject, on which the inspection of dead bodies cannot throw much light, has hitherto been too much neglected by those who wish to refer every thing to the anatomical structure of the parts; the manner in which they view the alterations which these parts manifest after death, has caused them to overlook those subtle and intricate changes, in which even the principles of vitality are immediately concerned. Such an omission has doubtless contributed to the uncertainty and want of accuracy so observable in the works of most pathological anatomists, and which even, at the present day, render them rather curious collections, less calculated to enlighten men of science than to dazzle the half learned. Observations have not however been wanting, in order to establish the changes which occur in the properties and functions of our organs; and if these be not sufficiently numerous to enable us to class them with accuracy, it is owing to the prevailing opinion, that they are necessarily connected with alterations of form and structure, and that it was proper to search for the causes of such changes, not by carefully observing the phenomena of the organs during life, but on an inspection of the degradations produced in them after death.

It is however possible, that by a singular concurrence of circumstances, certain organs become capable of exercising properties, and performing functions foreign to their nature, and which even belong to very different organs. Those parts of the animal body in which these dispositions or qualities occur, are evidently transformed and changed, though their new state does not coincide with the relative alterations in the system of their organization. If rare and singular facts did not always inspire me with distrust, I might adduce that extraordinary transposition of of hearing and sight from their natural organs, to the orifice of the stomach, so that sounds and colours excite in it the same sensation as they usually do in the eyes and ears.

Dr. Petelin has minutely described a similar transposition of the senses in several cataleptic females. A young lady of the department of Ardeche, who came to Montpelier, about five years ago, in order to consult the physicians of that place respecting an hysterical affection, complicated with cataleptic symptoms, exhibited an example of this singular phenomenon. She experienced during the continuance of these attacks such a high degree of sensibility about the præcordial region, that all the senses seemed to be concentrated in it alone. She referred to the stomach instead of the eye and ear, all the impressions produced by visual and sonorous bodies. This phenomenon occurring in a highly interesting young person, was not more an object of attention to physicians than of curiosity to the public. I cannot dissemble that such occurrences being in apparent opposition to all the known laws of nature, ought not to be received but with the greatest caution, and only after the most rigorous investigation. But if we multiply observations of this kind, if we state accurately the circumstances of each case, we must acknowledge the possibility of a phenomenon, which only, perhaps, appears to us marvellous, from the want of a sufficient number of similar facts, with which it may be compared. It would not be, however, difficult to multiply observations of a similar kind, at least, so far as regards the sense of hearing, of which the exercise requires not perhaps so much as the sight, an apparatus of a determinate structure; because the impressions made by sound depend on the tremors and vibrations of the air, which may strike with effect all the parts of the human body.

Haller mentions a young man, who in consequence of a nervous disease, acquired such a degree of sensibility, that all the organs of his body became as it were ear, and could distinguish, like that organ itself, the force and action of sounds. No physiologist is now ignorant, that parts which in a natural state are different both in sensibility and irritability, may acquire them under certain circumstances, which, however, neither change their texture nor composition.

The organs lodged in the internal cavities of the body much more readily assume new functions and properties than those which occupy its external surface. The organization nization of these last being more fixed and invariable is so much the more strictly connected with the properties and functions which belong to them. Their mutual dependence is so great, that the organs of the senses and the external membranes cannot experience any change in their structure, which does not at the same time influence the mechanism of their action. As the qualities and operations of the viscera, glands, and the internal organs are less dependent on any peculiar mode of structure, it cannot seem extraordinary that these organs should frequently supply each other's place. In this way the urethra and vagina have been known to supply the place of the rectum in those individuals, in whom this intestine was either obstructed or preternaturally contracted. The stercoraceous matters in such cases, instead of escaping by the anus, proceeded through an opening into these canals, and were thus evacuated. A more singular substitution is that of the secretory for other organs, which, though differing in structure from them, yet furnish the matter usually separated by the former, when continuing to discharge their functions. The urine, instead of being discharged in the usual way, is sometimes diverted from its natural course, and evacuated by the cutaneous pores, the pulmonary, mammary, the salivary and other organs.

Were it necessary, it might be readily proved that there is no secretory organ from which different humours have not been separated, and as little changed, as if furnished by the very organs destined to secrete them. Nature sometimes selects organs very remote from the uterus, to throw off the redundant menstrual blood. Thus the vessels of the nose, the eyes, the mammæ, the anus, the stomach, the lungs, &c. have all by turns furnished the catamenial flux. Hence it appears, that the properties and functions of the organs may be changed and transformed as well as the organic structure, the chemical composition, and the physical qualities of these parts. But we have not yet investigated this new class of transformations with the same accuracy and minuteness as the three former.

